# WAAR (World Alliance against Antibiotic Resistance): Safeguarding antibiotics

**DOI:** 10.1186/2047-2994-1-25

**Published:** 2012-07-09

**Authors:** Jean Carlet, Claude Rambaud, Céline Pulcini

**Affiliations:** 1 WAAR, 9 rue de la Terrasse, 94000, Creteil, France; 2 LIEN, 24 rue de Silly, Boulogne-Billancourt, 92100, France; 3 CHU de Nice, Service d’infectiologie, Nice, France; 4 Université Nice-Sophia Antipolis, Faculté de médecine de Nice, Nice, France

## Abstract

**Summary:**

Resistance to antibiotics has increased recently to a dramatic extend, and the pipeline of new antibiotics is almost dry for the five next years. Failures happen already for trivial community acquired infections, like pyelonephritis, or peritonitis, and this is likely to increase. Difficult surgical procedures, transplants, and other immunosuppressive therapies will become far more risky. Resistance is mainly due to an excessive usage of antibiotics, in all sectors, including the animal one. Action is urgently needed. Therefore, an alliance against MDRO has been recently created, which includes health care professionals, consumers, health managers, and politicians. The document highlights the different proposed measures, and represents a strong consensus between the different professionals, including general practicionners, and veterinarians.

## 

After saving countless lives, antibiotics are in danger of losing their effectiveness[1]. The reason is an alarming increase in bacterial resistance combined with a decline in the antibiotic pipeline flow. Treatment failures are already occurring in patients with infections whose only unusual feature is causation by multidrug-resistant (MDR) bacteria, some of which are capable of resisting all the available antibiotics [2]. These failures will become increasingly common, and some of them will result in life-threatening situations. In addition, risks will increase sharply in patients who require procedures associated with a high risk of infections (e.g., major surgery, organ transplantation, and immunosuppressive treatments).

Antibiotics are unique medications in that their targets (bacteria) are living organisms capable of adapting by developing mechanisms that confer resistance to antibiotics (e.g., mutations and the acquisition of resistance-gene vehicles). Despite this feature shared by no other medication, antibiotic prescription is viewed as a trivial act, both in humans and in animals. Limited improvements have been achieved, but France remains among the heaviest consumers of antibiotics in Europe.

Clearly, there is a pressing need to protect the effectiveness of antibiotics (and of anti-infectious medications in general) via proactive strategies similar to those used to save endangered species, in keeping with the concept of sustainable development. Antibiotics are unique medications and must be prescribed only with good reason. Each prescription should be thought carefully, and the short-term benefits to the patient (which obviously deserve priority in the event of a bacterial infection) should be weighed against two categories of deleterious effects: short-term adverse effects on the patient – such as manifestations of intolerance or allergies, which are unacceptable if the antibiotic is not indispensable – and negative medium-term effects on bacterial ecology, with selection of MDR organisms. Selected MDR organisms subsequently cause infections in the community, of which the patient is a member. The prescription of antibiotics must be the result of a complex deliberative process, whose components must obey evidence-based rules. This objective of achieving rational antibiotic prescribing practices requires an orchestrated effort by both the healthcare system users and the prescribers. Other strategies should be implemented concomitantly, such as campaigns to promote immunizations known to exert antibiotic-sparing effects (e.g., against influenza, measles, and pneumococcal pneumonia).

The World Alliance against Resistance to antibiotics (WAAR) is a crosscutting action plan designed by a small group of professionals and by the patient-support group LIEN to deal with the current emergency. This plan involves healthcare providers (in hospitals and private practice), veterinary medicine professionals, and the food industry. It is of immediate significance for current and future users of the healthcare system, as well as for all citizens.

The Alliance is a group of about 350 individuals representing all the key stakeholders including healthcare system users (LIEN, CISS, Patients for Patient Safety [WHO], and the Association for the defense of Victims of Nosocomial Infections [ADVIN] in Quebec). The scientific committee is composed of 80 international physicians of considerable renown. The Alliance receives support from 50 learned societies or professional groups in France and throughout the world. It is a nonprofit organization (French law of 1901) open to professionals and users worldwide.

SAFEGUARDING ANTIBIOTICS requires an orchestrated effort carried out jointly by healthcare system users and prescribers (in the broad sense of the term). Therefore, WAAR has chosen as its primary objective to raise awareness among all stakeholders of the urgency and magnitude of the threat. WAAR must lobby actively for antibiotics beyond the circle of insiders in order to raise awareness among policy-makers, international health organizations (WHO, World Organization for Animal Health, European Centre for Disease Prevention and Control), and the entire population. The Alliance, in France is designed to serve as a complement to two French nationwide programs, the Antibiotic Alertness Program for 2011–2016 and the Antibiotic Resistance Minimization in Veterinary Medicine Program. The Alliance has no conflicts of interest.

WAAR advocates a number of immediate measures:

a much more cautious and controlled approach to the use of antibiotics, in all areas;

relentless efforts to prevent the cross-transmission of MDR organisms both in hospitals and in private practice;

the expansion of basic and applied research efforts in human and veterinary medicine;

the development of new antibiotics, including via the identification of methods based on antiadhesion molecules to combat specific bacterial species;

the development of antibiotics or treatment strategies for veterinary medicine that have the smallest possible ecological impact;

the development of new vaccines;

a much more widespread use of diagnostic tests, with the goal of limiting the prescription of antibiotics to proven bacterial infections;

a ban of broad spectrum antibiotic use without an attempt to diagnose the cause of the infection

increased surveillance of antibiotic resistance and use, with regular feedback to healthcare professionals and the public;

education and training programs for healthcare professionals and consumers;

the rapid implementation of antibiotic programs for humans and animals, by making the necessary resources available.

Some of these measures are part of the Antibiotic Alertness Program for 2011–2016 implemented by the French National Health Agency (*Direction Générale de la Santé*, DGS) and of the Antibiotic Resistance Minimization in Veterinary Medicine Program. However, the Alliance advocates much stronger measures to be implemented on an emergency basis, most notably regarding the modalities of antibiotic prescription.

## Detail of the measures advocated by the Alliance

### A vast information campaign

A vast information campaign on antibiotics targeting physicians, veterinarians, pharmacists and consumers is needed to raise awareness of the threat to the life-saving potential of antibiotics. Consumers must be reminded that antibiotics have no effect on viruses, which account for the overwhelming majority of respiratory tract infections. Both physicians and consumers should feel accountable whenever an antibiotic is prescribed. The French public health insurance system (*Caisse Nationale d’Assurance maladie*, CNAM) is currently conducting an information campaign. This measure needs to be intensified, and information efforts must be thought through and developed in partnership with the key stakeholders, in a manner that is consistent with other components of the strategy for safeguarding antibiotics. The information campaign targeting healthcare professionals and consumers should include advice on basic hygiene and on the prevention of cross-transmission. These two topics should be dealt with in a well-orchestrated manner.

### Prescribing antibiotics in a reasoned manner, in hospitals and in private practice, in humans and in animals

#### Structural measures

Hospital physicians with specific training in antibiotic stewardship should be given a greater role. A decree is needed to specify their status, mission, and training; appropriate ratios; and funding modalities.

Infectious-diseases and medical-microbiology networks linking private practice physicians and hospitals should be created. Centers supplying advice on antibiotic treatment and ensuring the surveillance of bacterial resistance patterns in private practice, hospitals, and medical-social institutions should be set up as part of these networks, in connection with a reference hospital (similar to Medqual in the Loire region, Antibiolor in Lorraine, and Primair in Franche-Comté). Each center should have a telephone hotline for hospitals and another for private practices. Both hotlines should be operated by paid professionals. Thus, each region of the country should have a network of well-trained antibiotic stewards who can provide advice by telephone around the clock 7 days a week to healthcare facilities that have no antibiotic stewards on their staff.

Awareness of these networks and antibiotic stewards should be increased via vast communication campaigns targeting professionals, and incentives to encourage the use of these networks should be provided.

The entire process of antibiotic prescription and delivery to each patient identified by name should be entered into secure databanks.

Decision-making aids complying with suppress french recommendations should be widely disseminated (e.g., http://www.antibioclic.com).

A number of antibiotics should be reserved for use in humans (e.g., carbapenems and new antibiotics developed for humans), and restrictions should be placed on the use in animals of antibiotics that are critical to humans (cephalosporins and fluoroquinolones) (Reference 26 of the veterinary medicine program).

A study should be conducted to evaluate the prescription and delivery of antibiotics by veterinarians and the consequences on antibiotic consumption. European directives must be developed promptly to revise marketing practices related to the promotion and sales of antibiotics (reference 29 of the veterinary medicine program).

A vast program of university training and continuing education for professionals should be set up. Curricula for human and animal healthcare professionals should give priority to the wise use of antibiotics and to good hygiene practices, most notably those designed to limit the cross-transmission of microorganisms. In these areas, measures designed to modify current practices should receive recognition (P4P?).

The European program e-bug (http://www.e-bug.eu) designed to raise awareness of these objectives among schoolchildren should be widely advertised.

#### Technical measures

Each hospital should have a list of antibiotics whose use must be approved by an antibiotic steward (although the first few doses would be available immediately to avoid treatment delays)

Each antibiotic prescription to hospital patients should require validation by a senior physician.

An attempt to isolate a microbiological pathogen needs to be undertaken for all patients that receive broad spectrum antibiotics

Generic drugs should be used only if they have been proven as effective as the corresponding proprietary drugs.

Preference should be given to antibiotics that have limited ecological effects, and the most recent antibiotics should be used rationally.

Antibiotic treatment durations should be kept as short as possible, and when appropriate antibiotics should be stopped after a careful reappraisal (based on the clinical course and new results from the microbiology laboratory).

Antibiotics should be delivered only in the amount prescribed (if need be, by delivering only part of the contents of the package) to minimize self-medication; this measure has already been adopted in other countries.

A specific prescription form should be developed for antibiotics at high risk for inducing ecological effects, such as third-generation cephalosporins, fluoroquinolones, and carbapenems.

The measures taken for human medicine should be adapted to veterinary medicine, based on the specific features of animal care. Routine prophylactic antibiotic treatment of livestock and fish must be prohibited, except when the presence of risk factors has been documented. After the onset of an outbreak in a farm, early prophylactic antibiotic treatment of all animals or metaphylactic antibiotic treatment of selected subgroups may be in order. Only the amount of antibiotics corresponding to the prescription should be delivered. The treatment should be brief, in order to minimize its ecological impact. The price or price margin of veterinary antibiotics should be set according to the benefits they produce.

### A measure of considerable symbolic impact would consist in having the UNESCO include antibiotics on the World Heritage list

Effective antibiotics are an endangered species, and their use should be subsumed to ecological principles and integrated within a global sustained development concept. Because they of considerable universal value, antibiotics deserve to be protected.

### Preventing cross-transmission and selection pressure

#### Cross-transmission

The health threats due to fecal contamination with MDR bacteria require vigorous action. Under everyday conditions, person-to-person transmission of bacteria occurs chiefly via the hands. Hand hygiene practices must be improved in the community across the range of social activities. For example, individuals must learn to sneeze and cough into the crook of the elbow if immediate hand disinfection is not possible. The use of hydroalcoholic hand rubs should be encouraged in healthcare settings and, under some circumstances, in the community.

Hospitalized patients known to carry MDR bacteria should be isolated. In addition, patients with risk factors such as multiple hospital admissions, medical evacuation from abroad, and hospital admission abroad within the past year should be screened and isolated until the results are available. The best course of action may vary across MDR organisms. More generally, strict adhesion to standard hygiene recommendations is crucial with all patients.

In livestock farms, good hygiene and appropriate facilities and practices are indispensable. Hygiene is critically important wherever animals are raised.

#### Minimizing ecological effects

Waste products from hospitals and livestock farms should be processed appropriately. There is a need for rethinking waste treatment plant operations in order to prevent contamination of soil and water, as antibiotics continue to select MDR organisms in the natural environment.

Resources should be made available to improve the detection of resistant bacteria in drinking water and in foods such as meat and fish.

#### Supporting research

Intensified research efforts are needed to elucidate the epidemiological mechanisms underlying bacterial resistance and to develop new antibiotics, new vaccines, and non-antibiotic anti-infectious agents for use in human and veterinary medicine. Research into the behaviors that explain the greater consumption of antibiotics in France compared to other European countries may help to design effective campaigns targeting prescribers and the public, with the goal of improving antibiotic usage.

Financial incentives should be created to support startup companies focusing on the development of new products, as well as pharmaceutical companies exhibiting a firm commitment to a policy of good antibiotic usage.

### Improving the diagnosis of bacterial infections

Existing rapid diagnostic tests (RDTs) should be used widely and appropriately. They include the rapid strep test for streptococcal pharyngitis and urinary dipstick tests. In infections of greater severity, such as pneumonia, the C-reactive protein or procalcitonin level can be measured in a sample of capillary blood collected at the bedside (although the results must be confirmed). In many cases, these rapid and simple tests can rule out a bacterial infection, thereby avoiding the use of antibiotics. They should be reimbursed by statutory health insurance systems. Support should be provided to the development of tests characterized by greater specificity and sensitivity, as well as of multifunction tests.

All these crucial measures are being advocated by professionals, and professionals should be in charge of control processes and training. If these measures are not taken now, then the near future may witness the implementation on an emergency basis of compulsory measures that may be either inappropriate or difficult to accept.

### Enhancing and exploiting surveillance data

Europe has an outstanding system for monitoring antibiotic resistance patterns and antibiotic use. However, further surveillance efforts are needed in the community and in veterinary medicine. Furthermore, surveillance data should be communicated in simple terms and at regular intervals to healthcare professionals, decision-makers, and the public, both for the entire country and for each region.

### Evaluating the program

The objective of the French nationwide program for humans and animals is an at least 25% decrease in antibiotic consumption over 5 years.

WAAR advocates a 30% decrease over 3 years, in both humans and animals, to rapidly reach the mean value for European countries.

In conjunction with hygiene practices in hospitals and in the community (schools, other institutions, and families), this measure would be expected to stabilize resistance levels at their current values or perhaps even to obtain a decrease.

WAAR advocates the following resistance indicators and target values: penicillin-resistant pneumococci (target, 1%), methicillin-resistant *Staphylococcus aureus* (MRSA; target, 10%), *Escherichia coli* resistant to third-generation cephalosporins (target, <10%), glycopeptide-resistant enterococci (VRE) (target, sporadic cases), and carbapenem-resistant Enterobacteriaceae (CRE) (target, sporadic cases) this could vary according to countries.

The profile and amount of antibiotics prescribed by each physician working in office practice is monitored in France by the statutory health insurance system. The data thus obtained should provide a tool for physician self-evaluation and quality-indicator development. However, the monitoring procedure needs to be sharpened, in particular by factoring in the profile of each physician’s patient caseload. One approach may be to have professional organizations conduct critical reviews of random samples of patient files.

## Conclusion

To safeguard the small number of antibiotics that are still effective, and to protect the few antibiotics that will be introduced in the future, awareness must be raised throughout the country, on an emergency basis. Prescribers and consumers should work together to achieve this objective.

JOIN US NOW, by contacting

Jean Carlet, president of WAAR, 9 rue de la terrasse 94000 Créteil, FRANCE

jeancarlet@gmail.cofm.

Your help is vital!!

## This article was published in French in the journal *La Revue du Praticien* on May ….2012

## 

Members of the Alliance: Laurent Aaron, Jacques Acar, Mansour Adéoti (Secretary of the RIPAQS), Corinne Alberti, Serge Alfandari, Antoine Andremont, François Angouluant, Djillali Annane (Dean of the Paris Ouest University), Gilles Antoniotti, Jean-Pierre Aquino, Anne-Marie Armentera De Saxcé (Director of the Rothschild Foundation), Anne Arnera-Carlet, Pascale Arnould (President of the French Society for Primary Care), Pascal Astagneau (Director of the Paris North CCLIN), Claude Attali, Fréderic Auber, Jean-Pierre Aubert, Elisabeth Autret-Leca, Elie Azoulay, Françoise Ballereau, Gérard Bapt (representative of the Haute Garonne district), Géraldine Bardon, Jean Pierre Bedos, Sibylle Belivacqua, Fethi Bensalem, Nelly Beon André, Patrick Berche (Dean of the Paris 5 University), Frederique Bergheau, Gilles Berrut, Philippe Berthelot (of the French Society for Hospital Hygiene), FrederiqueBeuhourry-Sassus, Edouard Bingen, Jacques Birgé, Sandra Biscardi, Marie-Claude Bongrand, François Bourdillon, Alain Bousquet-Melou, Jean Brami, Stephane Bretagne, Christian Brun-Buisson, Fabrice Bruneel, Yves Bur (representative of the Bas Rhin district), Sandrine Buscail, Christelle Cabon, Emmanuelle Cambau, Philippe Caranco, Camille Carlet, Florian Carlet, Jean Carlet, François Caron (president of the ACAI), Alain-Michel Ceretti, Pascale Chaize, Martin Chalume Allier district), Pierre Charbonneau, Christian Chavanet, Jean Chastre, Jean-Daniel Chiche (president-elect of the European Society of Intensive Care Medicine), Christian Chidiac, Fabrice Chopin, Olivier Chosidow, Patrick Choutet, André Cicollela (in charge of the Health Division of the Green political party), Robert Cohen, Bruno Coignard, Catherine Cordonnier, René Courcol, Aline Creuwels, Vincent Dacquet, Laurent Degos (outgoing president of the French National Authority for Health), Valérie Delbos, Jean-François Delfraissy (Director of the ANRS and of the ITMO for infectious diseases), Pierre Dellamonica, Frederic Delille, Marie-Claude Demachy, Corinne Denis, Marie-Helène Denninger, Jean Claude Desenclos, Nicole Desplaces, Jean-François Dhainaut (Director of the High Commission on Biotechnologies), Isabelle Dijols-Lecuyer, Than Doco-Lecompte, Jean Doucet, Pierre-Louis Druais (president of the College of Primary-Care Physicians), Catherine Dumartin, Marie-Françoise Dumay, Michel Dupon (president of the College of Infectious and Tropical Diseases University Physicians), Jacques Fabry, Bruno Fantin, Albert Faye, Nathalie Floret, Sandra Fournier, Irène Frachon, Bertrand Gachot, Jacques Gaillat, Tatiana Galperine, Karine Gambarotto, Lisa Garcia, Bernard Garo, Anne Gaschet, Remy Gauzit, Gaetan Gavazzi, Louise Gazagne, Chistian Ghasarossian, Jacques Gilquin, Olivier Goeau-Brissonniere (president of the Medical Specialties Organization), Fred Goldstein, Alix Greder, Amandine Grain, Benoit Guery, Catherine Guignabert, Loic Guillevin (president of the French Society for Internal Medicine), Laurent Gutmann, Joseph Hajjar, Rodolphe Halama, Olivier Hanon, Yves Hansmann, Isabelle Hau, Veronique Hentgen, Jean-Pierre Hermet (user), Celine Hernandez, Laurent Hocqueloux, Bruno Housset, Sophie Hubiche, Benoit Huc, Françoise Ichou (writer), Vincent Jarlier, Dominique Jean, Laurent Jouffroy (president of the French Society for Anesthesia and Critical Care), Karine Kadri, Axel Kahn (geneticist, president of the Paris Descartes University), François Kidd, Pierre Kilidjean (user), Serge Kouzan, Jean-Philippe Lacour (president of the French Society for Dermatology), Matthieu Lafaurie, Thierry Lavigne, Olivier Lehiani, Jean-Patrick Lajonchere (director of the Paris St Joseph Hospital Group), Jérome Larché, Gérard Laroussinie, Peggy Larroude, Anne-Marie Lavenaire, Thierry Lavigne, Christine Lawrence, Agnès Lefort, Guy Lefrand (representative of the Eure 1ec district), Hervé Le Louet, Alain Lepape, Joel Leroy, Xavier Lescure, Pierre Lombrail (president of the French Society for Public Health), Matthie Lorrot, Olivier Lortholary, Anne Lotthe, Jean Christophe Lucet, Jean-Yves Madec, Alexandra Mailles, Jacky Maillet, Jean Luc Mainardi, Alain Manuguerra, Carole Marchand, Bruno Marchou, Emmanuelle Martin, Claude-Denis Martin, Alain Martinot, Patrice Massip, Sophie Matheron, Nathalie Maubourget, Thierry May, Olivier Meunier, Noel Milpied (president of the French Society for Bone Marrow Transplantation), Christelle Miquel, Benoit Misset, Jean Michel Molina, Philippe Montravers, Gérard Moulin, Pascale Moulin, Gilbert Mouthon, Cécile Mourlan, Marie-Hélène Nicolas-Chanoine (president of the Onerba), Gérard Nitenberg, Patrice Nordmann, Jocelyne Ouanich, Bernard Page, Pierre Parneix (director of the South-West CCLIN), Olivier Patey, Yves Pean, Martine Peres, Pascal Perez, Christian Perronne (president of the French Federation of Infectiologists), Dominique Peyramond, François Philippart, Marie-Laure Pibarot, Marie-Cecile Ploy, Anny Poursinoff (EELV representative of the Yvelines district), Philippe Pucheu (Director of the Diaconnesses Croix St Simon Hospital), Celine Pulcini, Christian Rabaud (president of the French Society for Infectious Diseases), Claude Rambaud (president of the LIEN), Bernard Régnier, Jean Reignier (president of the Society for French-Speaking Intensivists), Vincent Renard (president of the National College of Teaching Primary-Care Physicians), Jean-Claude Reveil, Patricia Ribaud, Agnès Riche, Jérome Robert, France Roblot, Olivier Romain, Monique Rothan-Tondeur, Willy Rozenbaum, Genevieve Ruault, Laurence Safont, Julien Saison, Marie Christine Saux (president of the French Society for Clinical Pharmacology), Anne Savey (director of the South-East CCLIN), Catherine Schlemmer, Benoit Schlemmer (Dean of the Paris 7 University), Jean-Luc Schmidt, Martine Sinègre, Muriel Soulier-Majidi, Jean-Philippe Tabut, Fabienne Tamion, Pierre Tattevin, Soraya Terzaki, Laurent Thiriet, Jean-François Timsit, François Trémolières, Dominique Trivier, Michel Troadec, Jean-Paul Stahl, Garance Upham (member of the Board of Patients for Patient Safety, WHO), Bruno Valeyre (president of the Society for French-Speaking Pulmonologists), Dominique Valla, Emmanuelle Varon, Homero Vasquez, Nathalie Vauvarin, Agnes Vincent, Daniel Vittecoq (president of the AMM), Pascal Voiriot, Pierre Weinbreck (president of the National College for Infectious Diseases), Michel Wolff, Yazdan Yasdanpanah, Jean-Ralph Zahar, Daniel Zara Goni, and Jean-Marc Ziza

International Scientific Committee: Fekri Abroug (Monastir, Tunisia), Murat Akova (Ankara, Turkey), Fatma Amer (Egypt), Massimo Antonelli (Rome, Italy), Apostolos Armaganidis (Athens, Greece), Antonio Artigas (Barcelona, Spain), Fernando Baquero (Spain), Yaron Bar-Lavie (Tel Aviv, Israel), Thierry Calandra (Lausanne, Switzerland), Abdelfattha Chabib (Casablanca, Morocco), Annie Chalfine (Tel Aviv, Israel), Nathan Clumeck (Brussels, Belgium), Jonathan Cohen (UK), Peter Collignon (Australia), François Clergue (Geneva, Switzerland), George Dimopoulos (Athens, Greece), Georges Ducel (Geneva, Switzerland), Ricardo Durlach (Argentina), Naima Elmdaghri (Casablanca, Morocco), Dan Engelhard (Jerusalem, Israel), Annegret Franf-Karwautz (Austria), Petra Gastmeier (Berlin, Germany), Abdul Ghafar (India), Donald Goldmann (Boston, MA, USA), Herman Goossens (Antwerp, Belgium), Thomas Gottlieb (Concord, Australia), Manuel Guzman (Venezuela), Inge Gyssens (Nijmegen, The Netherlands), Fadi Haddad (Beirut, Lebanon), Stephan Harbarth (Geneva, Switzerland), Hakima Himmich (Casablanca, Marocco), Salih Hoçoglu (Diyarbakir, Turkey), Waleria Hryniewicz (Varsovia, Poland), Namita Jaggi (Haryana, India), Mitsuo Kaku (Sendai City, Japan), Igor Karpov (Belarus), François Kidd (Mons, Belgium), Badreddine Kilani (Tunis, Tunisia), Georges Khalil (Beirut, Lebanon), Oui-Chang Kim (Seoul, South Korea), Gabriel Levy Hara (Argentina), Elisabeth Heisbourg (Luxemburg), Pierre-François Laterre (Louvain, Belgium), Moi-Lin Ling (Singapore), John McGowan (Atlanta, USA), Dimitri Matamis (Salonica, Greece), Kamal Marhoum El Filali (Casablanca, Morocco), Shaheen Mehta (Cape Town, South Africa), Maria-Lisa Moro (Italy), Dilip Natwani (Dundee, Scotland), Babacar N’Doye (Dakar, Senegal), Steve Opal (Providence, RI, USA), Trish Perl (Baltimore, MD, USA), José-Arthur Paiva (Porto, Portugal), Mercedes Palomar (Barcelona, Spain), Didier Pittet (Geneva, Switzerland), Jean-Francois Pittet (city, AL, USA), Peter Pronovost (Baltimore, MD, USA), Jérome Pugin (Geneva, Switzerland), Rosana Richtman (Brazil), Vladimir Rudnov (Russia), Wing-Hong Seto (Hong Kong, China), Evelina Tacconelli (Italy), Falilatou Tchaline (Togo), Jordi Valles (Barcelona, Spain), Christian van Delden (Geneva, Switzerland), Ethan Rubinstein (Winipeg, Canada), Christina Vandenbroucke (The Netherlands), Jos van Der Meer (Nijmegen, The Netherlands), Homero Vasquez (Chile), Jean-Louis Vincent (Brussels, Belgium), Eric Vohr (Baltimore, MD, USA), Andreas Voss (Nijmegen, The Netherlands), Jeanine Wiener-Kronish (Boston, MA, USA), Emile Zein (Beirut, Lebanon), Josephine Zoungrana (Burkina Faso).

## Learned societies providing active support to the Alliance

-Société Française de Médecine Générale (SFMG), Société de Pathologie Infectieuse de Langue Française (SPILF), Société Française de Microbiologie (SFM), Société Française d’Hygiène Hospitalière (SF2H), Société Française de Santé Publique (SFSP), Société de Réanimation de Langue Française (SRLF), Société Française d’Anesthésie Réanimation (SFAR), Société de Pneumologie de Langue Française (SPLF), Société Libanaise de Médecine Interne (SLMI), Société Française de Greffe de Moelle (SFGM), Société Française de Gériatrie et Gérontologie (SFGG), Association Tunisienne de Réanimation (ATR), Société Tunisienne de Pathologie Infectieuse (STPI), Sociedale Paulista de Infectiologia-Brésil, Société Belge de Microbiologie Clinique (BVIKM/SBIMC), Société Marocaine de Maladies Infectieuses (SMMI), Société Française de Dermatologie (SFD), Australasian Society for Infectious Diseases (ASID), Australian Society for Antimicrobials (ASA), Austrian Antibiotic Stewardship Group, Hellenic Society of Intensive Care, Société Française de Médicine Interne (SFMI), and European Society of Intensive Care Medicine (ESICM).

## Organizations providing active support to the Alliance

Association Le LIEN, Association Le CISS, Fédération des Spécialités Médicales (FSM), Association pour la chimiothérapie anti-infectieuse (ACAI),Observatoire National d’épidémiologie de la Resistance Bactérienne aux antibiotiques (ONERBA),Collège National des Généralistes Enseignants (CNGE), Collège national de médecine générale (CNMG), Fédération Française de pneumologie (FFP), Groupe de Pathologie Infectieuse en Pédiatrie (GPIP), Collège des Enseignants de Maladies Infectieuses (CEMIT), Fédération Française d’Infectiologie (FFI), Collège National de Pathologie Infectieuse (CNPI), Institut Maurice Rapin (IMR), Medqual (F. Ballereau), Antibiolor (C Rabaud), Observatoire du Risque Infectieux en Gériatrie (ORIG, Réseaux de surveillance des antibiotiques et des bactéries multi-résistantes du Sud Est), Le Forum des bio-hygiénistes, Arab Alliance for a prudent use of antimicrobials (Ar-Apua), Association Phagespoir, Programme National de lutte contre l’infection nosocomiale (PRONALIN, Senegal), Réseau International pour la Planification et l’Amélioration de la Qualité, et de la Sécurité dans Services de Santé en Afrique (RIPAQS), Infection Prevention and Control African Network (IPCAN), Association des médecins coordonnateurs en EHPAD, Association de Lutte contre les Infections Associées aux Soins (ALIAS), Ligue Africaine des Associations pour la Sécurité des patients (LIASEP), Portuguese alliance for the preservation of the antibiotic, ESGAP working group (ESCMID), and Association des victimes d’infection nosocomiale (ADVIN).
